# Facial Attractiveness Ratings from Video-Clips and Static Images Tell the Same Story

**DOI:** 10.1371/journal.pone.0026653

**Published:** 2011-11-11

**Authors:** Gillian Rhodes, Hanne C. Lie, Nishta Thevaraja, Libby Taylor, Natasha Iredell, Christine Curran, Shi Qin Claire Tan, Pia Carnemolla, Leigh W. Simmons

**Affiliations:** 1 ARC Centre of Excellence in Cognition and Its Disorders & School of Psychology, University of Western Australia, Perth, Western Australia, Australia; 2 School of Psychology, University of Western Australia, Perth, Western Australia, Australia; 3 Centre for Evolutionary Biology & School of Animal Biology, University of Western Australia, Perth, Western Australia, Australia; Istituto di Neuroscienze, Italy

## Abstract

Most of what we know about what makes a face attractive and why we have the preferences we do is based on attractiveness ratings of static images of faces, usually photographs. However, several reports that such ratings fail to correlate significantly with ratings made to dynamic video clips, which provide richer samples of appearance, challenge the validity of this literature. Here, we tested the validity of attractiveness ratings made to static images, using a substantial sample of male faces. We found that these ratings agreed very strongly with ratings made to videos of these men, despite the presence of much more information in the videos (multiple views, neutral and smiling expressions and speech-related movements). Not surprisingly, given this high agreement, the components of video-attractiveness were also very similar to those reported previously for static-attractiveness. Specifically, averageness, symmetry and masculinity were all significant components of attractiveness rated from videos. Finally, regression analyses yielded very similar effects of attractiveness on success in obtaining sexual partners, whether attractiveness was rated from videos or static images. These results validate the widespread use of attractiveness ratings made to static images in evolutionary and social psychological research. We speculate that this validity may stem from our tendency to make rapid and robust judgements of attractiveness.

## Introduction

Much has been learned in recent decades about what makes faces attractive and why we have the preferences we do. We know that averageness, symmetry and sexual dimorphism (especially femininity in female faces) are attractive, that these traits can signal important aspects of mate quality, and that they are preferred in sexual partners (for reviews, see [1]–[4]). We also know that attractiveness affects many social outcomes, with more attractive people generally receiving more favourable treatment [5].

A potential limitation of this work is that it is based on ratings made to static images of faces, usually photographs, which provide limited information about appearance. Moreover, several studies have reported non-significant correlations between attractiveness ratings made to static images and dynamic video clips of the same faces [6]–[8]. These results suggest that attractiveness may be judged differently in static and dynamic images and raise questions about the validity of attractiveness research based on ratings of static images.

There are, however, reasons for caution in accepting these reports of non-significant correlations. First, they are inconsistent across studies. Rubenstein [8] found no significant correlation for female faces (no male faces were shown), whereas Lander [6] and Penton-Voak and Chang [7] both found substantial, significant correlations for female faces, but not for males faces. Second, the non-significant correlations were generally positive and small-moderate in size (Pearson's r's from 0.19 to 0.38, except for male faces in Lander [6]), so that lack of significance could reflect limited power (48 faces in Rubenstein [8], 24 per sex in Lander [6], 20 per sex in Penton-Voak & Chang [7]). Third, the static images were single frames taken from videos, which can look odd, unless carefully selected to represent possible static configurations (eg., resting pose). Finally, two studies have reported strong and significant agreement between attractiveness ratings made to videos and static images, one using photographs [9] and the other using carefully selected freeze frames [10].

More generally, we suggest that ratings of attractiveness from static images cannot be completely invalid because they accurately signal important aspects of mate quality such as genetic heterozygosity [11], and they predict real-life success in attracting sexual partners (e.g., [12]). Nevertheless, the lack of agreement between attractiveness ratings of videos and static images in the studies cited above is a cause for concern. Moreover, little is know about the components and consequences of attractiveness rated from videos and whether they resemble those reported previously for attractiveness rated from static images.

The broad aim of the present study was to determine how confident we can be about the validity of an attractiveness literature that is based largely on static images of faces. To do so, we first asked how well facial attractiveness ratings made to videos agree with ratings made to static images. We used independent groups of raters to ensure there were no carry-over effects and used carefully selected video frames for the static images to ensure natural-looking faces. Our videos contained more information about 3-dimensional face structure (by showing the face from different viewpoints) than those used previously, thus allowing greater opportunity for disagreement. Second, we asked whether the components of video-rated attractiveness are similar to those reported previously for static images. Would averageness, symmetry and sexual dimorphism, which are attractive in static images (for a meta-analysis see [4]), also be attractive in videos of faces. Finally, we asked whether video-rated attractiveness has similar consequences for mate choice to static-attractiveness. If attractiveness plays a role in human sexual selection, as is widely assumed, then more attractive males should have greater mating success, which in the absence of contraception would be associated with higher reproductive success. We examined three commonly used measures of mating success: total number of sexual partners, number of short-term partners (less than one month) and age of first sexual intercourse. The first two measures are likely to be highly correlated, but it can be useful to consider number of short-term partners separately, because women's short-term partner choices are likely to be more strongly affected by male appearance than their longer-term partner choices.

To address these questions we made 10-second video-clips of 60 male faces. Each video displayed the face in multiple viewpoints (from left profile through front-view to right profile), at rest, counting (muted for ratings) and smiling. For each video, we selected a front view of the face at rest, with a neutral expression, closed mouth and direct gaze, for use as the static image. These frames were taken from parts of the video where the face was not moving and looked like normal photographs. The males also completed demographic and sexual history questionnaires and a scale measuring attitudes to sexual behaviour. These measures were completed in private, labelled with a personal identity number, and inserted into locked boxes. Ensuring anonymity in this way promotes honest responding [13]. Independent groups of female participants (individually) rated the videos on attractiveness, averageness (reverse-scored distinctiveness), symmetry and masculinity. Following previous studies, distinctiveness was rated rather than averageness, because “very average” is often used to mean unattractive (e.g., [11], [12]). Female raters were used because opposite sex ratings are more relevant for assessing the consequences of attractiveness for mating success.

## Methods

### Ethics

This research was approved by the Human Research Ethics Committee at the University of Western Australia. All participants provided informed written consent.

### Participants

Sixty heterosexual, Caucasian adult males, aged 17 to 35 years were video-taped for use as stimuli. None had facial hair (beards or mustaches). Fifty-eight heterosexual, Caucasian adult females, aged 17–35 years, participated as raters. All participants were recruited from the University of Western Australia community or from the families and friends of the experimenters. They received course credit or $10 for participating.

### Stimuli and Measures

#### Videos and static images

The male participants were videotaped individually, seated in a small room with symmetrical lighting. A Canon digital video camcorder (HDV 1080i- HV20) was used, at a fixed distance of 1.8 m from the subject's face, at eye level. The videos showed the head and shoulders against a grey background, with a black drape covering clothing. Each video depicted the following sequence of actions: (1) rotate head from left profile throught center to right profile (without turning body), (2) face the camera directly and count aloud (from 7 to 13), and (3) smile. Participants were instructed to maintain a neutral expression from events (1) to (2). A metronome set at one beat/second was used to maintain a consistent speed for the head views (1 view/beat) and counting (2 counts/beat). The final videos were edited to 10 seconds and muted, using iMovie 4.0. Still frames of each of male face (front-view shot with neutral expression, mouth closed and eyes looking directly at the camcorder) were extracted from the videos, to create a set of static images. The complete stimulus set consisted of 60 videos and the 60 corresponding static images (hair visible). All faces were displayed for rating on a Macintosh OS X Laptop and viewed from 57 cm. They measured approximately 17°×13°.

#### Self-reported sexual behaviours and sexual attitudes

After the video session, the male participants completed a questionnaire, reporting age, sex, sexual orientation, number of short-term (less than one month) sexual partners, total number of sexual partners and age of first sexual intercourse. We also asked about sexual infidelity (cheating or poaching), but only 12 participants reported any infidelities, so this variable was not examined further. Finally, attitudes towards sexual relationships were assessed using a short questionnaire used in previous studies [12], [14], [15]. Participants indicated agreement (1 = *strongly agree*, 9 = *strongly disagree*) with four statements about sexual relationships: ‘Sex without love is ok’, ‘Casual sex outside of existing relationships is OK’, ‘Sex on the first date is OK’ and ‘I would need to know my partner emotionally and psychologically before having sex’. The responses were summed (with the final statement reverse-scored) to produce a composite attitude score (CAS), where higher scores indicate more conservative attitudes. This measure has moderate internal consistency and high test-retest reliability [12]. Participants were assured that their responses would completely anonymous and confidential. They were only identifiable by a 4-digit personal identification number (i.e., they created their own code and wrote it down on the cover of the questionnaires). All questionnaires were completed in private and inserted into a locked box.

#### Appearance ratings

Female raters were randomly assigned to rate the 60 videos on either attractiveness (n = 13), distinctiveness (n = 12), symmetry (n = 11) or masculinity (n = 9). Distinctiveness ratings were reverse-scored to provide a measure of averageness. The 60 videos were shown for 10 secs each in random order, after 3 practice trials (showing different faces from the test faces). Another 13 females rated the 60 static images on attractiveness. These images were shown for 3 secs each, because the judgements are likely to be made quickly [16] resulting in frustration if asked to wait 10 secs before responding. All ratings were made using 10-point Likert scales (e.g., 1 = *extremely unattractive*, 10 = *extremely attractive*) using labelled keyboard keys. Participants were encouraged to use the full range of the scale. At the end, raters were asked if they knew any of the males shown, and if so which ones, so that these could be excluded from further analysis. All raters were tested individually and a chin-rest was used to ensure a fixed viewing distance of 57 cm.

## Results

A mean rating was calculated for each face, on each variable, by averaging relevant scores across raters (excluding ratings for known faces - on average, fewer than three per rater) (Table 1). For video ratings, there was excellent consensus on attractiveness (Cronbach's α = .92) and good consensus on averageness (Cronbach's α = .77), symmetry (Cronbach's α = .85) and masculinity (Cronbach's α = .84). As expected, there was also excellent consensus on attractiveness rated from static images (Cronbach's α = .92). All the appearance variables were normally distributed.

**Table 1:** Descriptive statistics for appearance ratings, mating success variables, attitudes and age.

Variable	*N*	*M* (*SD*)	*Mdn* (Range)
Video-attractiveness	60	4.1 (1.2)	3.9 (1.8–7.2)
Static-attractiveness	60	4.1 (1.1)	4.1 (1.9–6.5)
Video-averageness	60	5.3 (1.4)	5.3 (1.8–7.5)
Video-symmetry	60	5.2 (1.2)	7.6 (2.0–7.3)
Video-masculinity	60	5.8 (1.5)	5.8 (2.9–9.3)
Total number of sexual partners	60	3.5 (3.9)	2 (0–17)
Short-term sexual partners	60	1.7 (2.6)	1 (0–13)
Age at first sex	48^1^	18.1 (2.8)	18 (14–26)
Sexual Attitudes (CAS)	60	21.0 (6.7)	21.0 (8–36)
Age	60	21.8 (4.2)	20.5 (17–35)

1 virgins excluded.

### Agreement between Video-Attractiveness and Static-Attractiveness

**Figure 1:**
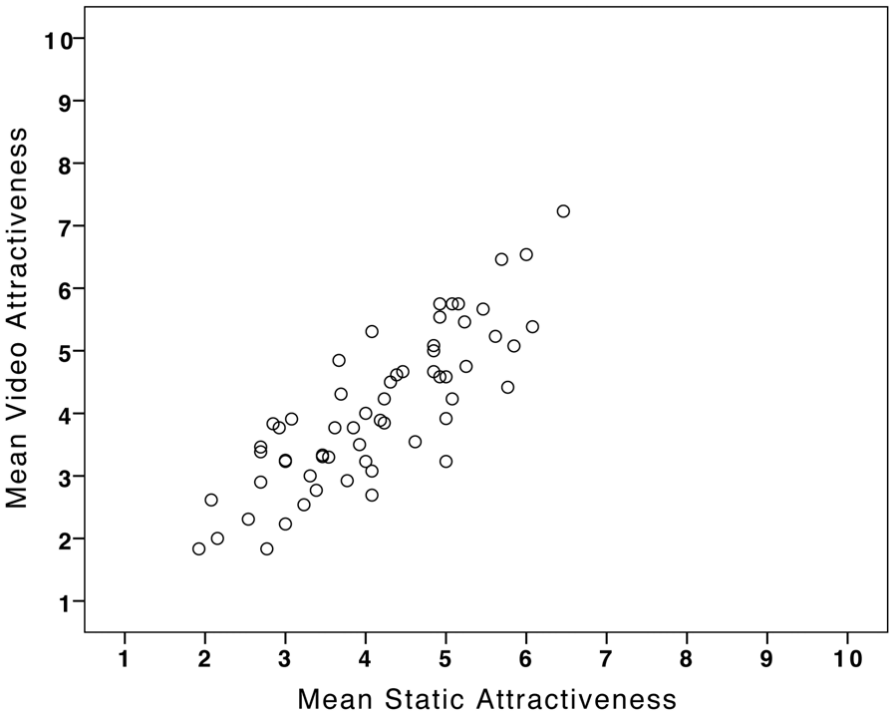
The association between attractiveness rated from videos and static images of faces (N = 60).

Importantly, there was strong agreement between attractiveness rated from videos and static images, r = 0.83, N = 60, p<.001 (Figure 1). This agreement is particularly high given that the videos and static images were rated by independent groups and that the videos contained additional information about 3-dimensional facial structure (appearance from different viewpoints), movement (during speech) and expression (smiles). The distributions of the two kinds of attractiveness ratings were also very similar, with no significant difference in mean ratings, t<1 (Table 1).

### Components of Attractiveness

Video-rated attractiveness correlated significantly with averageness, r = 0.38, p<.01, symmetry, r = 0.36, p<.01, and masculinity, r = 0.30, p<.05 (all n's = 60) (also rated from the videos). These correlations are very similar to those reported in a meta-analysis of studies using photographs: mean r's of 0.40 for averageness, 0.23 for symmetry, and 0.35 for masculinity [4]. Clearly, these traits are attractive in both videos and static images of faces.

### Consequences: Mating Success

Two of the mating success variables, total number of sexual partners and number of short-term partners, were positively skewed and could not be transformed to normality. Therefore, we used nonparametric Generalized Linear Modelling (GZLM) to test whether video-attractiveness predicts those variables. We controlled for age, which was positively correlated with these variable, and sexual attitudes, which were negatively correlated with these variables (more conservative attitudes associated with fewer partners) (Table 2). Age of first sex was slightly positively skewed due to two extreme cases, but plotting the residuals of a standard multiple regression model against predicted values produced a random scatter plot. Therefore, we used multiple regression to test whether video-attractiveness predicts age of first sex (removing the two extreme scores did not change the pattern of results). We again controlled for age and sexual attitudes. For completeness and comparison purposes, we conducted similar analyses using static-attractiveness as the predictor variable.

**Table 2:** Correlations between attractiveness, mating success variables, sexual attitudes and age.

Variables	1	2	3	4	6	7	8
1. Video-attractiveness		.83***	.27*	.30*	−.21	−.11	−.14
2. Static-attractiveness	.63***		.10	.18	−.26	.01	−.33*
3. Total sexual partners	.13	.01		.89***	−.21	−.47***	.39**
4. Short-term sexual partners	.14	.05	.77***		−.12	−.48***	.31**
6. Age of first sex	−.09	−.17	−.21	−.12		.17	.52**
7. Sexual attitudes (CAS)	−.06	.03	−.46***	−.48***	.20		−.19
8. Age	−.07	.22	.39***	.33***	.38***	−.13	

*p<.05,

**p<.01,

***p<.001.

Pearson product-moment correlation coefficients (r) are shown above the diagonal and non-parametric Kendall's rank-order correlations (τ) are shown below the diagonal. All *N's* = 60 except for those with Age at first sex (*n* = 48).

For the Generalized Linear Modelling (GZLM) we used negative binomial distributions with a log-link. These provided the best fit (smallest values of AIC, AICC, BIC). A separate model was tested for each outcome variable: total number of sexual partners, short-term partners. Video-attractiveness was the main predictor variable of interest, with age and sexual attitudes entered as covariates. The models yield Exp(B) scores, which indicate how much the outcome variable changes with each unit increase in the predictor variable [17]. Because it is the exponentiated product of a linear equation, Exp(B) represents the multiplicative effect (as opposed to an additive effect) of a unit increase in the predictor variable, when all other predictors are held constant [18]. An Exp(B) value of 1 indicates that there is no relationship between the predictor and the outcome variable. An Exp(B) value >1 denotes a positive relationship, and an Exp(B) value <1 denotes a negative relationship.

As can be seen from Table 3, video-attractiveness was a significant positive predictor of both total number of sexual partners and number of short-term sexual partners, after adjusting for the effects of attitudes and age. Very similar results were obtained for static-attractiveness, as expected given the high correlation between the two measures. There were very similar Exp(B) values, and highly overlapping confidence intervals for the two models (although the effect of static-attractiveness on total numbers of partners did not quite reach significance). As expected, age and sexual attitudes were also significant predictors of numbers of partners. There was no evidence of over-dispersion in any of the models (i.e., deviance ratios >1), with deviance ratios ranging from 0.656 to 0.765.

**Table 3:** Video-attractiveness and static-attractiveness as predictors of mating success (number of sexual partners), controlling sexual attitudes (CAS) and age, using GZLM (*N* = 60).

Sexual partners	Attractiveness	Predictors	B (SE)	*p*	Exp(B)	95% CI for Exp(B)
Total	Video	CAS	−.10 (.02)	.001	.91	(.87, .94)
		Age	.12 (.03)	.001	1.13	(1.06, 1.20)
		**Attractiveness**	**.20 (.10)**	**.037**	**1.22**	**(1.01, 1.48)**
	Static	CAS	−.10 (.02)	.001	.91	(.87, .94)
		Age	.13 (.03)	.001	1.14	(1.07, 1.22)
		**Attractiveness**	**.17 (.11)**	**.108**	**1.19**	**(.96, 1.47)**
Short-term	Video	CAS	−.16 (.03)	.001	.86	(.81, .90)
		Age	.14 (.04)	.001	1.15	(1.07, 1.24)
		**Attractiveness**	**.28 (.11)**	**.012**	**1.32**	**(1.06, 1.64)**
	Static	CAS	−.16 (.03)	.001	.85	(.81, .90)
		Age	.16 (.04)	.001	1.17	(1.09, 1.26)
		**Attractiveness**	**.33 (.13)**	**.010**	**1.39**	**(1.08, 1.79)**

The attractiveness results are highlighted in bold.

The results for age of first sex are shown in Table 4. Neither video- nor static-attractiveness predicted age of first sex for the sexually active men in our sample (n = 48). These results are similar to those reported previously for attractiveness rated from photographs [12]. The present results suggest that this previous failure to find a relationship between attractiveness and age of first sex is not an artifact of using static images to rate attractiveness.

**Table 4:** Video-attractiveness and static-attractiveness as predictors of age at first sex, controlling sexual attitudes (CAS) and age, using multiple regression (*N* = 48).

Attractiveness	Predictors	B (SE)	*p*	beta	95% CI for B
Video	CAS	.09 (.04)	.039	.27	(.01, .18)
	Age	.31 (.07)	.001	.56	(.17, .45)
	**Attractiveness**	**−.20 (.24)**	**.398**	**−.11**	**(−.68, .27)**
Static	CAS	.10 (.04)	.031	.28	(.01, .18)
	Age	.30 (.07)	.001	.54	(.16, .45)
	**Attractivess**	**−.19 (.27)**	**.496**	**−.09**	**(−.73, .36)**

The attractiveness results are highlighted in bold.

## Discussion

We found very high agreement between attractiveness ratings made to videos and static images. This result is particularly striking because our videos contained much more information than our static images. In addition to showing the face at rest and speaking, and with neutral and smiling expressions, they showed a full range of views (from left profile rotating through front-view to right profile), which has not been done previously [6]–[10]. Clearly, good agreement despite these differences provides strong support for the validity of assessment ratings made to static images.

Not surprisingly, given this high agreement, we also found that video-attractiveness ratings had similar components and consequences to those reported previously for ratings made to static images. Averageness, symmetry and masculinity were all significant components of video-attractiveness, as found previously for static-attractiveness (for a review and meta-analysis, see [4]). In addition, men with higher video-attractiveness ratings were more successful in obtaining sexual partners than their less attractive peers, as found previously for men with higher static-attractiveness ratings [12]. Clearly, these results validate the use of attractiveness ratings from static images in evolutionary and social psychological research.

The dynamic images used here provided a lot of additional information that could potentially be used to modify assessments of attractiveness made to the static images. Indeed the correlation of .83 between attractiveness ratings from videos and static images obtained here, although very high, still leaves 31% of the variance that could be affected by this additional information. Nevertheless, even with additional information about 3-dimensional face shape, expression variation and face mobility during speech, attractiveness judgements based on videos yielded very similar components and very similar consequences for mate choice, to attractiveness judgements based on photographs. These results are consistent with a recent report that the appeal of sexual dimorphism does not vary between videos and static images of faces [19]. We also found no difference in the overall level of rated attractiveness for videos and static images. Even the presence of smiles in the videos did not increase attractiveness ratings. This result is consistent with other evidence that smiles do not increase male attractiveness ([7], [20]; but see [21], [22]).

Attractiveness judgements are made very rapidly and do not change much with longer viewing times [16]. Our results suggest that they may also be resistent to change when additional information is presented. They are also consistent with findings that ratings made to different views (front versus profile) in static images show good agreement [23]. Overall, it seems that assessments of facial attractiveness are relatively robust to variations in the amount and type of visual information that we have about a face.

Our finding that more attractive males were more successful in obtaining sexual partners provides further behavioural evidence that physical attractiveness is important in human sexual selection (cf., [12]). Moreover, attractive males obtained more short-term partners than their less attractive peers, consistent with women's self-reports that they value physical attractiveness in short-term sexual partners [24]. Because males can increase their reproductive success (in the absence of contraception) by having more partners, our results suggest that both attractive traits and female preferences for such traits, may be sexually selected. Furthermore, attractive traits like averageness, symmetry and masculinity can signal important aspects of mate quality (for reviews, see [1]–[4]), so that preferences for these traits may be, at least in part, adaptations for finding high quality mates.

Our findings have some limitations. First, we only used male faces and Rubenstein's [8] original report of no significant correlation between video- and photo-attractiveness was for female faces. However, there is no theoretical reason to expect a different result for female faces, and others have reported substantial and significant correlations for female faces [6], [7], [9]. We suggest, therefore, that the results would be unlikely to differ for female faces. A second potential limitation is the use of ratings rather than measurements of averageness, symmetry and masculinity. The pros and cons of ratings versus measurements have been discussed extensively elsewhere [4], [12], [25], but there is good evidence for the validity of these trait ratings, which may even have advantages over current measurement methods for 2-dimensional images.

In summary, we found no evidence to suggest that research on the components and consequences of attractiveness based on ratings of single, static images, lacks validity. On the contrary, we found very strong agreement between ratings of attractiveness made to static images and to videos by two independent groups of raters. This agreement occurred even though the videos contained a lot more information than the photographs, about both the 3-dimensional structure and mobility (from neutral to smiling and during speech) of the faces, which could potentially affect perceptions of attractiveness. Nor was there evidence that different criteria were used to judge attractiveness in the two media, with averageness, symmetry and masculinity being rated as attractive in the videos as found previously for static images. Finally, regression analyses yielded very similar effects of attractiveness rated from videos and static images on success in obtaining sexual partners. We conclude that even though static images necessarily provide limited information about facial appearance, they may provide a valid measure of facial attractiveness. This validity may stem at least in part from the rapidity with which judgements of attractiveness are made, and their resistance to change [16], [26].
